# Effectiveness of bundled care in the management of cerebral hemorrhage complicated by pulmonary infection in China: A systematic review and meta-analysis

**DOI:** 10.1371/journal.pone.0312882

**Published:** 2024-11-01

**Authors:** Yamei Zhang, Xiulan Wu, Ming Lu, Lu Sun, Xibo Sun, Zulin Dou, Zhanhao Liu

**Affiliations:** 1 Guangdong Sanjiu Brain Hospital, Guangzhou, Guangdong, China; 2 Department of Nursing Department, Mingxin Rehabilitation Medical Center, Guangzhou, Guangdong, China; 3 Department of Rehabilitation Medicine, The Third Affiliated Hospital, Sun Yat-sen University, Guangzhou, Guangdong, China; Tabriz University of Medical Sciences, IR Iran, ISLAMIC REPUBLIC OF IRAN

## Abstract

**Objective:**

Aimed to systematically evaluate the therapeutic and rehabilitative effects of bundled care on patients with cerebral hemorrhage complicated by pulmonary infection in China.

**Methods:**

Relevant literature was retrieved from multiple databases and original studies investigated the efficacy of bundled care in managing cerebral hemorrhage complicated by pulmonary infection were included. Data analysis was conducted using Meta-analysis software (RevMan 5.3 and Stata 17.0). The Cochrane risk of bias assessment tool was used to evaluate the risk of bias. Sensitivity analysis was performed to evaluate the robustness of the results. Funnel plot, Begg and Egger test were conducted to assess the presence of publication bias. The protocol was registered in PROSPERO (CRD42023475738).

**Results:**

Twelve studies involving 1049 patients were included in this meta-analysis. The results showed that bundled care significantly reduced the duration of antibiotic use and pulmonary infection (SMD = -0.77), reduced the duration of tracheal intubation (MD = -5.35), and shortened hospital stay (MD = -6.30). The effective rate of treatment (OR = 8.39), satisfaction degree (OR = 5.65), anxiety (MD = -4.52) and depression scores (MD = -4.38), and quality of life scores (MD = 11.75) were improved after bundled care intervention compared to routine care. Assessment of publication bias showed no significant evidence of publication bias.

**Conclusions:**

Bundled care can significantly shorten the duration of illness treatment and hospital stay of patients with cerebral hemorrhage complicated by pulmonary infection, improve treatment effectiveness and satisfaction degree, alleviate anxiety and depression, and promote the improvement of quality of life.

## Introduction

Cerebral hemorrhage, an acute condition characterized by the rupture and bleeding of brain blood vessels, requires immediate treatment and comprehensive care to minimize further neurological damage and the risk of complications [1]. The incidence of cerebral hemorrhage is high, estimated at 12–15 cases per 100,000 people. In Western countries, cerebral hemorrhage accounts for 15% of strokes, while in China, it represents a higher proportion within strokes, ranging from approximately 18.8% to 47.6%. Additionally, cerebral hemorrhage presents a serious threat, with a high mortality rate within one month of onset, ranging from 35% to 52% [2,3]. Less than half of the patients are able to regain their ability for independent living within six months, imposing a significant medical burden on various countries [4]. Moreover, the occurrence of complications complicates the treatment and recovery of patients. Pulmonary infection is a common complication in patients with cerebral hemorrhage, with an incidence rate reaching 68%. Common symptoms include dyspnea, fever, and coughing, and severe cases can progress to sepsis, potentially leading to multiple organ failure [5,6]. Pulmonary infection not only complicates the recovery process but also poses a significant mortality risk, especially among elderly patients [6,7].

In recent years, the integrated care model, particularly bundled care, has gained significant attention and implementation in Chinese hospitals. By fostering interdisciplinary collaboration, adhering to standardized care protocols, and leveraging real-time data monitoring, bundled care aims to enhance the quality and efficacy of patient care [8,9]. In the context of managing cerebral hemorrhage complicated by pulmonary infection, bundled care has been adopted to improve treatment outcomes, shorten recovery periods, enhance patient quality of life, and reduce mortality rates [10].

Despite the proliferation of studies exploring the use of bundled care for managing cerebral hemorrhage complicated by pulmonary infection, a comprehensive evaluation of its effects remains notably lacking. This gap in the literature underscores the necessity for a thorough investigation into the efficacy of bundled care protocols in these complex clinical scenarios. Therefore, this study aims to conduct a robust meta-analysis that synthesizes existing research data, allowing for a nuanced understanding of how bundled care impacts patient outcomes in cases of cerebral hemorrhage complicated by pulmonary infection. By performing detailed data analysis, we aim to provide healthcare teams with compelling evidence to support the integration and implementation of bundled care in clinical practice, ultimately enhancing patient outcomes and care efficiency.

## Methods

### Data sources and searches

This meta-analysis followed the guidelines stipulated in the Preferred Reporting Items for Systematic Reviews and Meta-Analyses (PRISMA) [11]. A comprehensive search was conducted across multiple databases, including PubMed, Web of Science, EMBASE, Cochrane library databases, CINAHL, the China National Knowledge Infrastructure (CNKI), Chinese Science and Technology Periodical Database (VIP), WanFang Knowledge Service Platform, and Chinese Biology Medicine, encompassing articles published from the inception of these databases up until Feb 01, 2024. The search strategy included a combination of Medical Subject Heading terms, entry terms, and text words. Search terms involved “Stroke”, “Cerebrovascular Accident”, “Brain Vascular Accident”, “Cerebral Hemorrhage”, “Care”, “Nursing”, “Pneumonia”, “Pneumonitis”, “Pulmonary Inflammation”, “Lung Inflammation”. The search strategy for Chinese databases was adjusted according to medical terms used in the literature. Following the removal of duplicate records, the titles and abstracts of the remaining citations were screened to assess their potential for inclusion. Subsequently, full-text articles were thoroughly reviewed to determine eligibility according to the predefined inclusion criteria. The protocol for this meta-analysis was registered in PROSPERO under the registration number CRD42023475738.

### Inclusion criteria

Articles included in this meta-analysis were selected based on the participants, interventions, comparisons, outcomes, and study designs framework. (1) Participants: Patients with cerebral hemorrhage complicated by pulmonary infection. (2) Intervention: The experimental group received bundled care in addition to routine nursing, which primarily involved respiratory management, dietary management, position management, appropriate medication use, psychological care, and health education. (3) Control: The control group received nursing modalities other than bundled care, such as routine care and comprehensive care. (4) Outcome: Prognostic indicators of the disease, including the duration of antibiotic use and pulmonary infection, duration of endotracheal intubation, length of hospital stay, treatment effectiveness, satisfaction levels, anxiety and depression scores, and quality of life scores. Treatment effects were classified into three levels: a) cure: clinical signs disappeared after treatment, and lung infection-related tests returned to normal; b) improvement: clinical symptoms and related signs improved following treatment, and lung infection-related tests showed progress; c) ineffective: no significant change was observed in the patient’s condition after treatment. Treatment effectiveness was measured by the proportion of cases categorized as cured or improved. (5) Study design: Only randomized controlled trials were included, and all included studies were required to be conducted in hospitals within China.

### Exclusion criteria

Exclusion criteria were as follows: (1) Non-randomized controlled trials, retrospective analyses, or case reports. (2) Articles with no available data or duplicate date.

### Data extraction and quality assessment

Two independent authors (X.L.W. and Y.M.Z) conducted data extraction and assessed the risk of bias. If there was disagreement, a third author (M.L.) was consulted for resolution. The extracted data included publication year, first author, participants characteristics (age and sample size), intervention types, and outcome data. The risk-of-bias assessment for the included studies was performed using the Cochrane risk-of-bias assessment tool. The assessment covered random sequence generation, allocation concealment, blinding of participants and personnel, blinding of outcome assessment, incomplete outcome data, selective reporting, and other biases.

### Data analysis

RevMan 5.3 software (The Nordic Cochrane Center, The Cochrane Collaboration; London, UK) and Stata 17.0 software (StataCorp LLC, College Station, TX, USA) were used for data analysis. The Q statistic and the I^2^ statistic were used to assess the heterogeneity. For continuous outcomes, the effective size was expressed as the standardized mean difference (SMD) or mean difference (MD) with their corresponding 95% confidence interval (CI). Dichotomous outcomes were evaluated using risk ratios and 95% CI. A random-effects model was used for all analyses when heterogeneity was significant (I^2^ > 50%), while a fixed-effects model was employed when heterogeneity was low (I^2^ ≤ 50%). Statistical significance was set at P < 0.05. Forest plots were generated to illustrate the results of individual studies. Sensitivity analysis was performed by excluding each study to evaluate the robustness of the results. Funnel plot, Begg test, and Egger test were conducted to assess the presence of publication bias, with a P level of > 0.05 indicating no evidence of publication bias.

## Result

### Literature search and characteristics of included studies

**Fig 1:**
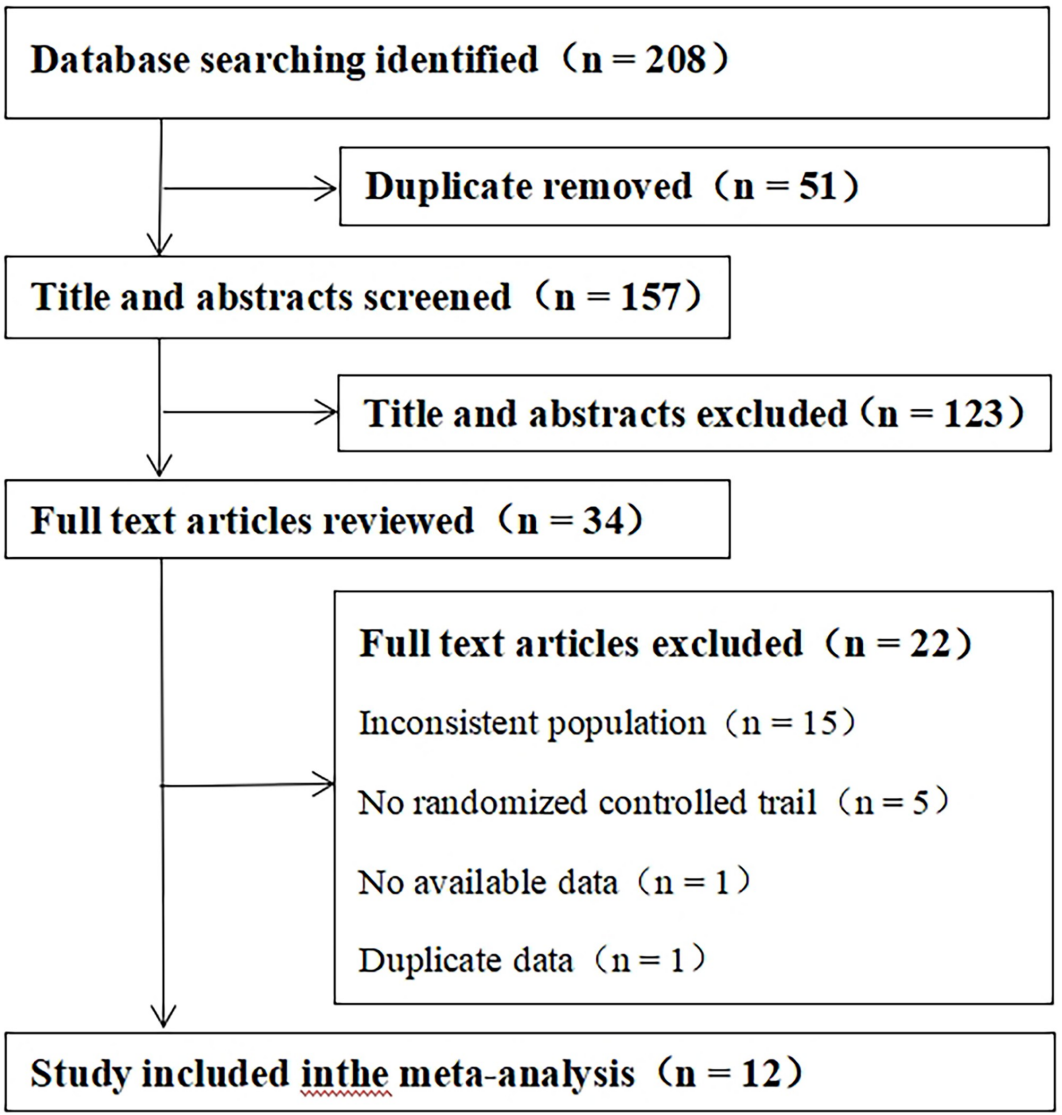
Study flowchart.

A total of 208 articles were retrieved. After screening and applying inclusion/exclusion criteria, 12 studies [12–23] were included in the meta-analysis, comprising a total of 1049 patients (524 in the experimental group and 525 in the control group). The flowchart of literature selection is shown in Fig 1, and the basic information of the included literature is presented in Table 1.

**Table 1:** Overview of studies included in the meta-analysis.

Reference	Country	No. of Participants		Mean Age (y)		Nursing Model		Outcome indicator
		Experiment Group	Control Group	Experiment Group	Control Group	Experiment Group	Control Group	
Jiang [12]	China	41	41	68.30±5.40	69.20±5.70	Bundled Care	Routine Care	①②③
Tao [13]	China	94	95	66.20±4.40	66.20±4.40	Bundled Care	Routine Care	④
Jiang [14]	China	41	41	68.72±3.01	69.04±3.22	Bundled Care	Routine Care	①②③④⑤
Luo [15]	China	30	30	73.80±3.20	73.80±3.20	Bundled Care	Routine Care	④⑤
Chen et al [16]	China	46	46	63.90±3.30	64.80±5.70	Bundled Care	Routine Care	①②③④
Liu [17]	China	33	33	68.55±8.53	66.54±6.31	Bundled Care	Routine Care	④⑥⑦⑧
Wang [18]	China	42	42	59.11±6.52	59.06±6.48	Bundled Care	Routine Care	①②③⑤
Huang [19]	China	28	28	64.50±5.00	64.90±5.30	Bundled Care	Routine Care	①③
Huang [20]	China	54	54	56.25±12.47	57.42±11.61	Bundled Care	Routine Care	①③
Xiao [21]	China	33	33	66.54±6.31	68.55±8.53	Bundled Care	Routine Care	⑥⑦⑧
Hua et al [22]	China	30	30	64.89±3.01	67.93±2.8	Bundled Care	Routine Care	④⑥⑦
Li et al [23]	China	52	52	65.79±4.75	65.53±4.42	Bundled Care	Routine Care	①②③⑤⑧

①the duration of antibiotic use and pulmonary infection; ②the duration of endotracheal intubation; ③the length of hospital stay; ④the treatment effectiveness; ⑤the satisfaction degree; ⑥the Self-Rating Anxiety Scale; ⑦the Self-Rating Depression Scale;⑧the quality of life scores.

### Meta-analysis results

#### The duration of antibiotic use and pulmonary infection

**Fig 2:**
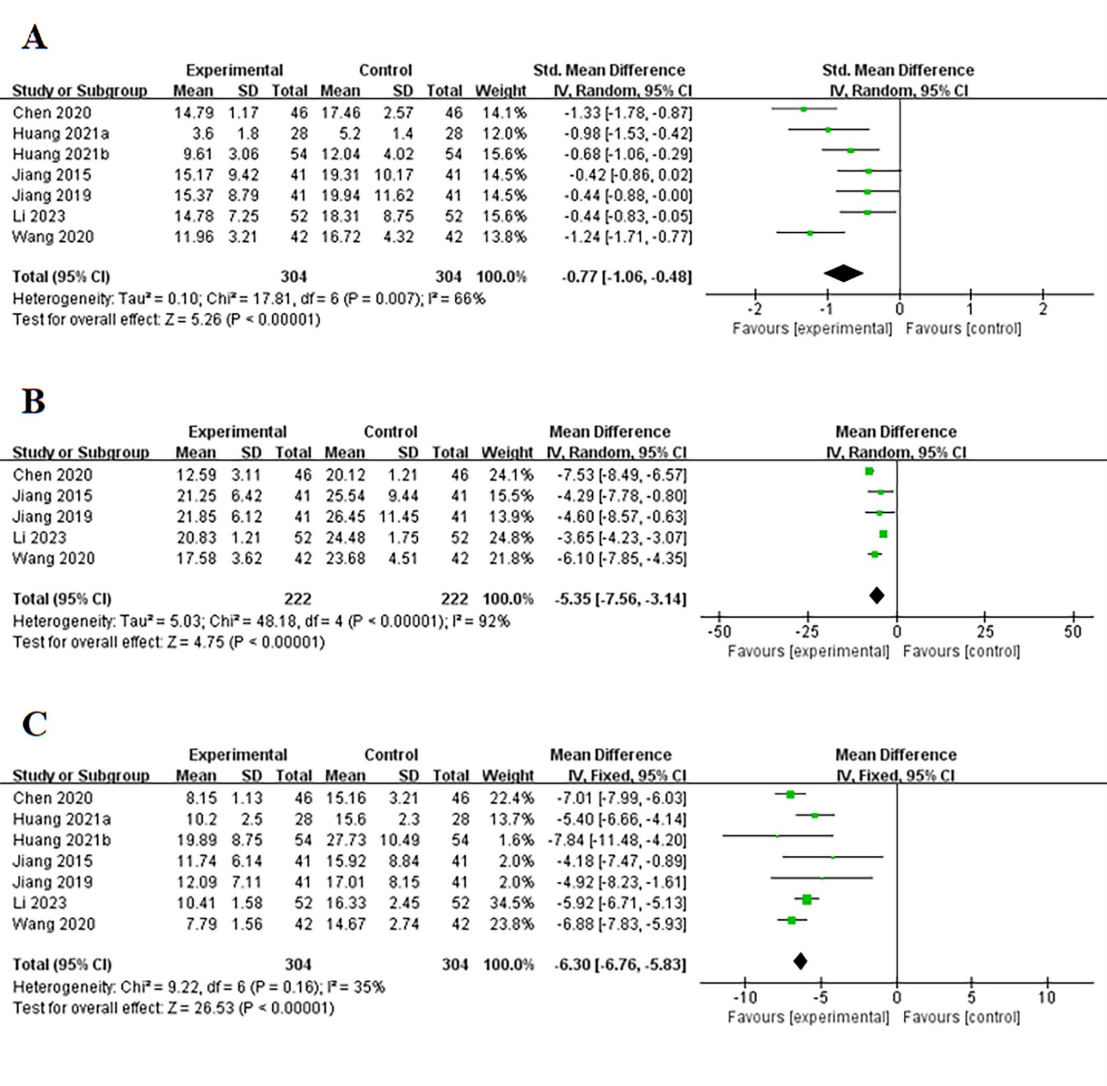
Forest plot of (A) the duration of antibiotic use and pulmonary infection, (B) the duration of endotracheal intubation, (C) the length of hospital stay.

Seven studies [12,14,16,18–20,23] reported the duration of antibiotic use and pulmonary infection. The results indicated that bundled care could significantly reduce the duration of antibiotic use and pulmonary infection in patients with cerebral hemorrhage complicated by pulmonary infections compared to the control group (P < 0.001), with high heterogeneity (I^2^ = 66%) (Fig 2A).

#### The duration of endotracheal intubation

Five studies [12,14,16,18,23] reported the duration of endotracheal intubation. The results showed that bundled care could help cerebral hemorrhage patients with pulmonary infections to be extubated earlier compared to the control group (P < 0.001), with high heterogeneity (I^2^ = 92%) (Fig 2B).

#### The length of hospital stay

Seven studies [12,14,16,18–20,23] reported the length of hospital stay. The results indicated that bundled care could significantly reduce the length of hospital stay compared to the control group (P < 0.001), with low heterogeneity (I^2^ = 35%) (Fig 2C).

#### Treatment effectiveness and satisfaction degree

**Fig 3:**
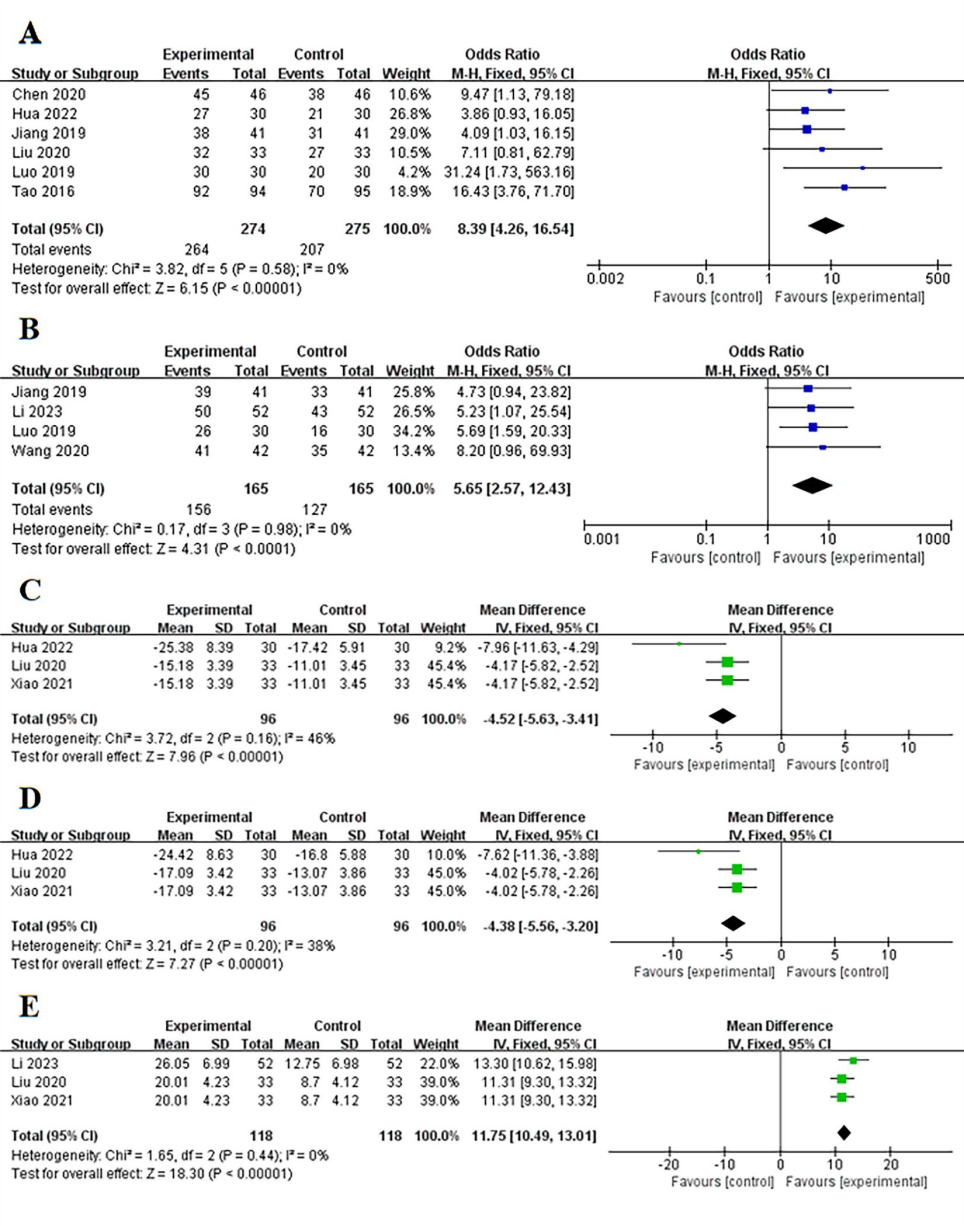
Forest plot of (A) the treatment effectiveness, (B) the satisfaction degree, (C) the Self-Rating Anxiety Scale, (D) the Self-Rating Depression Scale, (E) the quality of life scores.

Six studies [13–17,22] reported treatment effectiveness and four studies [14,15,18,23] reported satisfaction degree. The results showed that bundled care significantly improved treatment effectiveness and satisfaction degree compared to the control group (P < 0.001), with low heterogeneity (I^2^ = 0) for both (Fig 3A and 3B).

#### The anxiety / depression scores, and quality of life scores

Three studies each reported anxiety scores [17,21,22], depression scores [17,21,22], and quality of life scores [17,21,23]. The results indicated that bundled care could significantly reduce anxiety and depression scores and improve quality of life compared to the control group (P < 0.001), with relatively low heterogeneity (I^2^ = 38% / 46% / 0) (Fig 3C–3E).

### Sensitivity analyses and reporting bias

**Fig 4:**
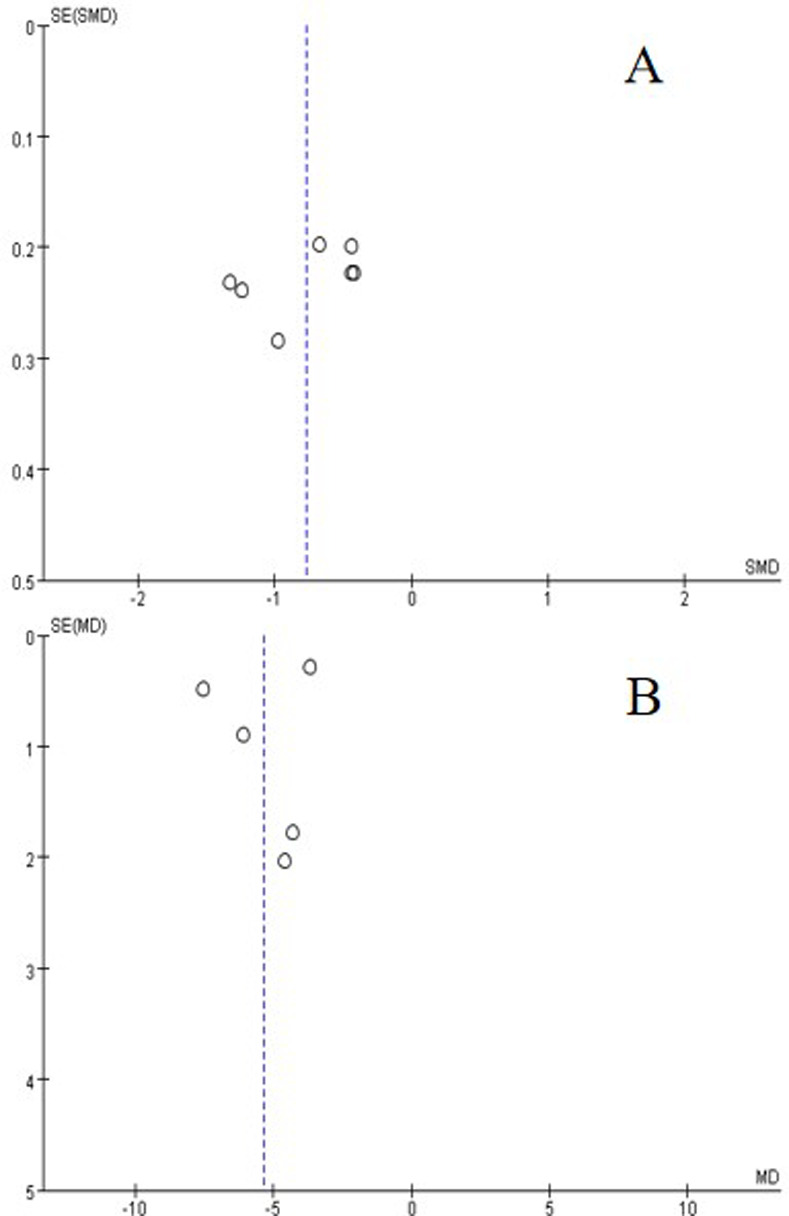
Funnel chart for publication bias. (A) the duration of antibiotic use and pulmonary infection, (B) the duration of endotracheal intubation.

Heterogeneity was high in the outcome measures of the duration of antibiotic use and pulmonary infection, and the duration of endotracheal intubation. Sensitivity analysis did not alter the overall impact of bundled care on study outcomes (Table 2). Funnel plots indicated publication bias in the duration of antibiotic use and pulmonary infection, and the duration of endotracheal intubation (Fig 4), but egger tests (P values of 0.058 and 0.082, respectively) and begg tests (P values of 0.099 and 1.000, respectively) suggested no publication bias.

**Table 2:** Sensitivity analysis results.

Outcome indicator	Study omitted	Estimate	95% CI	
the duration of antibiotic use and pulmonary infection	Wang 2020	-2.34	-2.95	-1.72
	Jiang 2015	-2.84	-3.81	-1.87
	Jiang 2019	-2.82	-3.78	-1.87
	Chen 2020	-3.10	-4.42	-1.78
	Huang 2021	-3.19	-4.04	-2.35
	Huang 2021	-3.07	-4.22	-1.91
	Li 2023	-2.85	-3.85	-1.86
	Combined	-2.88	-3.80	-1.96
the duration of endotracheal intubation	Wang 2020	-5.12	-7.84	-2.41
	Jiang 2015	-5.54	-8.02	-3.06
	Jiang 2019	-5.47	-7.91	-3.02
	Chen 2020	-4.57	-6.08	-3.05
	Li 2023	-6.33	-7.80	-4.85
	Combined	-5.35	-7.56	-3.14

### Quality assessment

**Fig 5:**
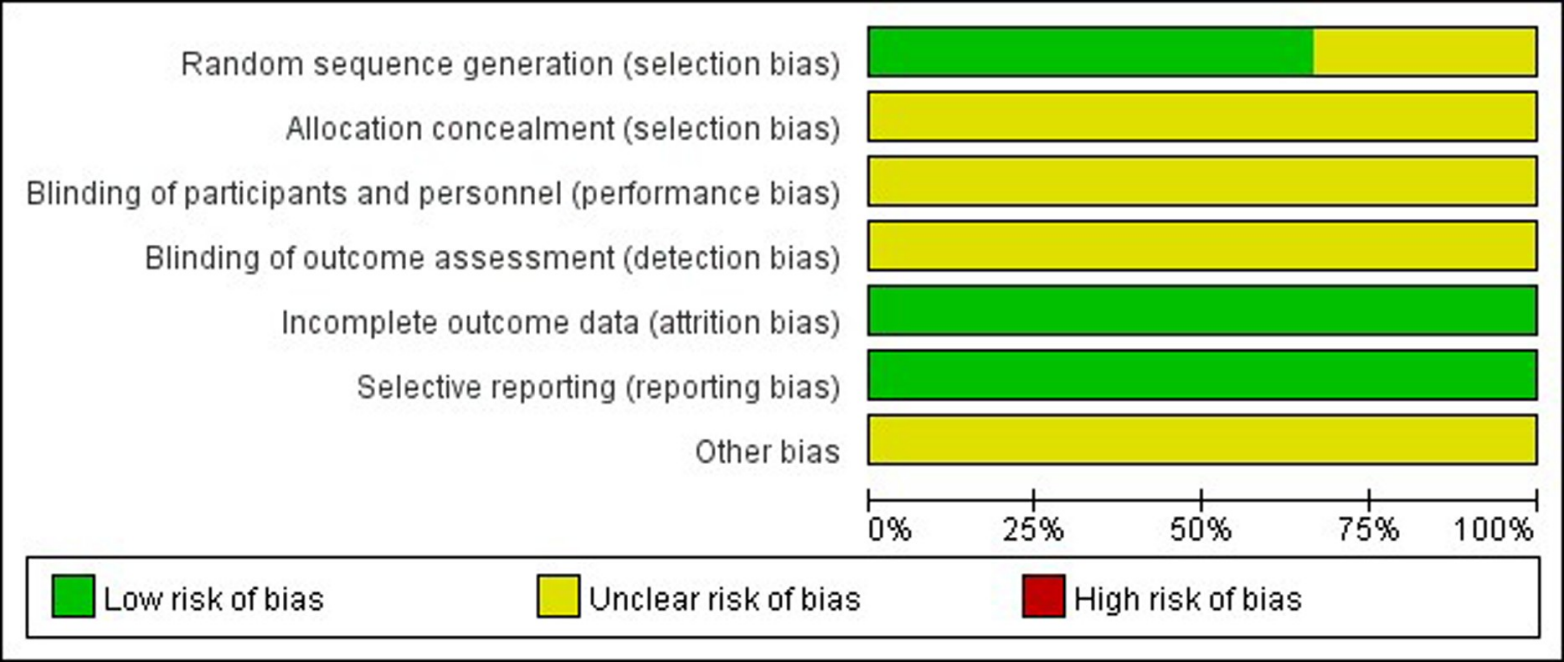
Risk of bias graph. The judgement of review authors about each risk of bias item presented as percentages across all included studies.

**Fig 6:**
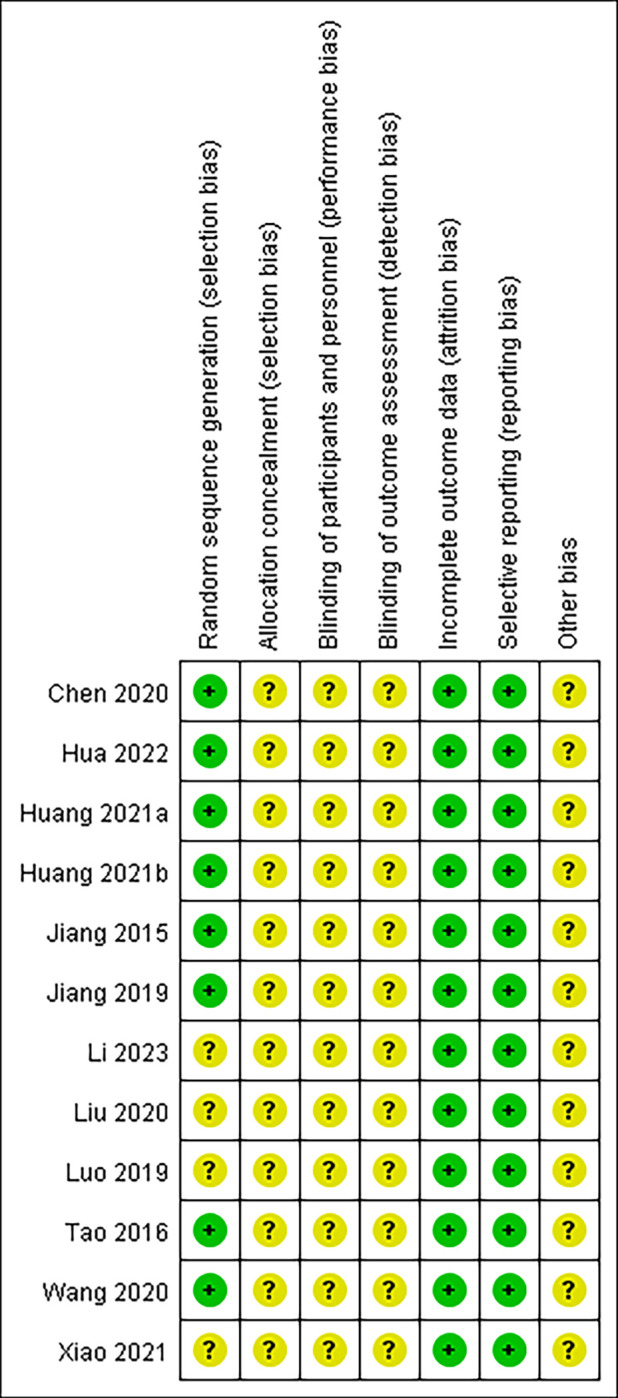
Risk of bias summary. The judgement of review authors about each risk of bias item for each included study.

The assessment of bias risk in included studies is shown in Figs 5 and 6. The overall quality of the included studies is considered to be low to moderate. Most studies did not provide adequate details on random sequence generation and allocation concealment.

## Discussion

Intracerebral hemorrhage complicated by pulmonary infection is one of the most common and serious complications of intracerebral hemorrhage, leading to deterioration of patients’ condition, heightened treatment complexities and risks, prolonged hospitalization, escalated medical expenses, and the potential development of complications such as respiratory failure, empyema, and lung abscess. These factors significantly impact patients’ health and quality of life. Within the treatment framework, the pivotal role of nursing staff is as critical as that of clinical physicians [3,6].

Bundled care, as a targeted nursing model, can optimize treatment outcomes and enhance patient satisfaction through comprehensive, coordinated, and efficient processes. This approach requires the medical team to assess the specific conditions of patients, evaluate disease status and treatment needs, develop personalized nursing plans, determine necessary nursing measures and schedules, integrate nursing measures for concentrated implementation, provide continuous and targeted nursing within a certain timeframe, make individualized adjustments and improvements to nursing plans based on patient responses and treatment progress, and evaluate and monitor nursing effectiveness [8,10]. This study represents the first meta-analysis-based investigation on bundled care for treating cerebral hemorrhage complicated by pulmonary infection, providing valuable theoretical and practical backing for managing this condition. Let’s now delve several key indicators.

Bundled care exhibited a positive impact on controlling the duration of pulmonary infections. Through a synthesis of multiple studies, it was evident that patients with cerebral hemorrhage who developed pulmonary infections and received bundled care experienced a significant reduction in the duration of antibiotic use and pulmonary infections. This signifies that bundled care not only diminishes the incidence of pulmonary infections [24,25] but also more effectively manages their progression and spread, thereby expediting the recovery process [6].

The outcomes of this meta-analysis revealed that patients under bundled care had notably shorter endotracheal intubation times compared to those in traditional care groups. This suggested that bundled care plays an important role in providing respiratory support, maintaining airway patency, and contributing to reduced endotracheal intubation duration. Consequently, this diminishes the reliance on mechanical ventilation and enhances respiratory function, aligning with findings from studies by Liu et al [26,27].

Moreover, bundled care demonstrated a favorable impact on hospital stay, treatment efficacy, and patient satisfaction within our meta-analysis. Pulmonary infection not only prolongs the hospitalization of patients with cerebral hemorrhage but also elevates in-hospital mortality rates [28]. Patients receiving bundled care effectively curtailed disease progression and complications through comprehensive and continuous care [29,30]. Consequently, bundled care leads to reduced hospital stay, improves treatment efficacy, and heighten patient satisfaction levels.

Additionally, bundled care positively influenced patients’ anxiety and depression scores, as well as their quality of life scores. Emotional disturbances and cognitive decline are prevalent issues following cerebral hemorrhage, which can impede patient compliance and treatment efficacy [31,32]. Patients receiving bundled care exhibited superior outcomes in anxiety and depression scores, alongside elevated quality of life scores. This underscores the ability of bundled care to offer detailed and personalized care, alleviate psychological stress, enhance mental well-being, and foster the recovery of both physical and mental health [33,34].

The literature included in this meta-analysis involves bundled care programs that consist of respiratory management, nutritional management, positional management, and appropriate medication. These measures not only accelerate the recovery from pulmonary infection but also enhance treatment efficacy and reduce the length of hospital stay. By strengthening health education, the occurrence of other complications can be prevented, further facilitating patient recovery. The reduction in hospital stay and the acceleration of the recovery process contribute to increased patient satisfaction with treatment. Additionally, enhancing psychological care for patients and providing psychological support helps to alleviate anxiety during hospitalization, thereby promoting the recovery of both physical and mental health. Multidisciplinary participation in bundled interventions has been shown to effectively reduce disease incidence and improve patient outcomes [35,36].

This study demonstrated the effectiveness of bundled care in the treatment of cerebral hemorrhage complicated by pulmonary infection through a meta-analysis. This may encourage clinicians to adopt the bundled care model more widely in daily practice, thereby improving treatment outcomes and quality of life for patients. Additionally, the study could promote relevant departments to formulate policies that support bundled care, facilitating its implementation in hospitals and healthcare institutions to better meet patients’ treatment needs. The findings of this study provide a foundation for future research, which can further explore the applicability of bundled care in different patient populations or care environments.

However, it is crucial to acknowledge certain limitations in this study. Firstly, the limited number and relatively low quality of included studies may impact the stability and generalizability of the results. Secondly, significant heterogeneity was observed for certain outcomes, and all included studies were conducted in China, which may introduce biases. Additionally, the study did not account for other potential intervention effects on the outcomes.

Hence, further research is imperative to fortify and validate the results of this meta-analysis, ascertain optimal practices and implementation strategies for bundled care, and establish more rigorous guidelines to offer comprehensive and effective clinical support for managing cerebral hemorrhage complicated by pulmonary infection. Moreover, the application of bundled care should consider individual patient variances and unique circumstances, necessitating appropriate adjustments and optimizations to achieve superior treatment outcomes.

## Conclusion

In summary, the application of bundled care has demonstrated significant effectiveness in the treatment of cerebral hemorrhage complicated by pulmonary infection in China. Beyond controlling the duration of pulmonary infection and endotracheal intubation, bundled care has also shown potential to reduce hospital stay, enhance treatment efficiency and patient satisfaction, and improve patients’ anxiety and depression scores, as well as elevate their quality of life. These findings underscore the crucial clinical value of bundled care in managing cerebral hemorrhage complicated by pulmonary infection.

## Supporting information

S1 Checklist PRISMA 2020 checklist. (DOCX)

S1 File (XLSX)
